# Use of dill extracts as a natural preservative on shelf‐life extension of rainbow trout croquettes during refrigerator storage

**DOI:** 10.1002/fsn3.3658

**Published:** 2023-09-01

**Authors:** Emre Caglak, Baris Karsli

**Affiliations:** ^1^ Faculty of Fisheries Recep Tayyip Erdogan University Rize Turkey

**Keywords:** dill extract, healthy fast food, natural protective, refrigerated storage, trout croquettes

## Abstract

In this study, changes in quality parameters of rainbow trout croquettes treated with dill extracts obtained by brewing (BDA) and distillation (DDA) methods were investigated during 61 days of refrigerated storage (+2 ± 1°C). Physicochemical, microbial, and sensory parameters of the rainbow trout croquettes were analyzed once every 4 days. It was observed that the precooking process and additives influenced the nutrient composition and color change of the croquettes. Total volatile base nitrogen and thiobarbituric acid values did not exceed the limit values in any group during refrigerated storage and these values were found as 23.10 ± 0.89 mg/100 g and 3.13 ± 0.08 mg MDA/kg in the control group (C), 21.00 ± 0.92 mg/100 g and 2.77 ± 0.14 mg MDA/kg in BDA group, and 21.70 ± 0.92 mg/100 g and 2.85 ± 0.07 mg MDA/kg in DDA group, respectively. Extract treatments, especially DDA, resulted in a significant reduction in the counts of total aerobic mesophilic and psychotropic bacteria in trout croquettes compared to the control group. It was determined that the sensory scores of all croquette groups decreased during storage and the acceptability scores of the C, BDA, and DDA groups reached on the 33rd, 57th, and 61st days of storage, respectively. According to the results, this work demonstrated that the dill extracts both distilled and brewed can be used as a natural additive to improve the quality and extend the shelf life of rainbow trout croquettes.

## Graphical abstract

It has been determined that dill extract obtained by different methods restricts microbiological growth, inhibits lipid oxidation, and improves sensory taste in refrigerated trout croquettes. The results showed that the addition of dill extract extended the shelf life by 24–28 days compared with the control samples. This work demonstrated that the dill extracts both distilled and brewed in can be used as a natural additive to improve the quality and extend the shelf life of rainbow trout croquettes.

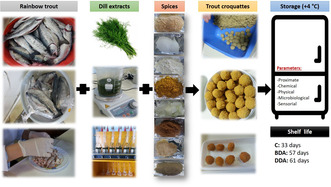

## 1 INTRODUCTION

Food culture is affected by the economic, social, and cultural changes of the period, and these changes directly or indirectly affect the nutritional habits of the society (Timmer, 2009). With the development of the food industry, many factors such as the support of mass production in food, the widespread use of ready meals, the increased participation of women in business life, the increase in population, urbanization, migration, the development of technology, the time lack for eating while working, changes in living conditions, and family structure have caused changes in food and culinary culture (Özpolat & Çoban, 2012). Thus, the resulting time constraints have made it inevitable for people to turn to ready‐to‐eat foods for practical nutrition (Önçel, 2015). In parallel with the increase in ready‐to‐eat food consumption, food manufacturers and marketers have recently needed new packaging and storage techniques or materials to support these processes in terms of the accessibility of these products all over the world. However, ready‐to‐eat foods are especially rich in carbohydrates and saturated fats (Çankırılıgil & Berik, 2017). For this reason, the content of ready‐made foods is very important in terms of consumer health. Seafood plays an important role in a healthy diet as a source of animal protein. In today's world, where the importance of healthy nutrition is increasing, the orientation toward healthy foods such as seafood has also increased (Keser & İzci, 2020). Seafood is a rich source of high polyunsaturated fatty acids, high protein, easily digestible protein, as well as it contains many vitamins and minerals. However, these products are among the perishable foods due to their biological structure. For this reason, it is necessary to store fishery products under suitable conditions and to apply appropriate processing techniques after fishing (Karsli & Caglak, 2021). In addition to the storage or packaging of seafood products without additives, there are also studies on offering them for consumption in different flavors and varieties.

Trout, which is one of the most widely cultivated species in Turkey, constitutes 35.12% (165,683 tons) of Turkey's aquaculture production in 2021 and 20.71% of the total production (TUIK, 2022), and it is marketed as live, fresh, chilled, frozen, smoked, and canned in Turkey and abroad (Keser & İzci, 2020). In addition, many products such as stuffed fish (Metin, 2003), paté (Ünlüsayın et al., 2007), fish doner kebab (Şimşek & Kiliç, 2011), croquettes (Berik et al., 2011; Çankırılıgil & Berik, 2017, 2018), fish balls (Keser & İzci, 2020; Öksüztepe et al., 2010), cake (İnanlı et al., 2011), puff pastry (Kaba et al., 2013), and caviar (Özpolat & Patır, 2009) were made from rainbow trout in order to increase product diversity and consume healthy food.

Since ancient times, medicinal and aromatic plants have been used to increase and maintain the taste, smell, aroma, and quality characteristics of foods, as well as their antiseptic and pain reliever properties (Maleš et al., 2022). The antioxidant properties of plants and spices have gained importance as an additive as well as being the subject of research, together with the chemical properties and differences in plants. In addition to being important for human health, antioxidant‐containing substances also play an active role in the quality parameters and shelf life of foods (Balıkçı & Yavuzer, 2018).

In recent years, antioxidant compounds have been widely used as coating or packaging materials. These compounds are applied to foods by spraying, mixing, and dipping, thus changing the taste and color of the products, affecting their acceptability by the consumer, as well as delaying spoilage with their antioxidant properties. In this context, it is a good option to include antioxidant components in packages to increase the shelf life of these products (Bhowmik et al., 2022). Oxidative balance is very important in maintaining the quality of foods containing high amounts of unsaturated fatty acids, such as seafood, and the most effective way to prevent or slow down oxidation in the degradation process is the use of antioxidant substances (Balıkçı & Yavuzer, 2018; Bensid et al., 2014). Antioxidants are divided into synthetic and natural. Today, the production and consumption of healthy food has become increasingly widespread, and in this context, synthetic antioxidants such as butylated hydroxyanisole (BHA), butylated hydroxytoluene (BHT), and propyl gallate (PG) have begun to leave their place to natural antioxidants (Kannaiyan et al., 2015). Thus, natural antioxidant and antimicrobial substances of plant origin attract the attention of both researchers and industry (Singh et al., 2005; Ucak & Fadıloğlu, 2020).

Dill (*Anethum graveolens* L.), a rich source of flavonoids, phenolics, tannins, saponins, terpene, and cardiac glycosides, is native to Mediterranean countries and Southeastern Europe (Oshaghi et al., 2015). Dill has been used to relieve gastrointestinal problems from ancient times. In addition, dill is useful in the treatment of colds, bronchitis, loss of appetite, stomach pain, urinary tract problems, hyperlipidemia, spasms, and seizures (Tizghadam et al., 2021). Dill, which has a rich essential oil content, is used in the fields of health, agriculture, and food with main compounds such as carvone and d‐limonene (Mutlu‐Ingok & Karbancioglu‐Guler, 2017; Singh et al., 2005). Antimicrobial (Jianu et al., 2012; Mutlu‐Ingok & Karbancioglu‐Guler, 2017; Rasheed et al., 2010; Singh et al., 2005; Tuchila et al., 2008) and antioxidant (Oshaghi et al., 2015) activates of dill extract and essential oil have also been reported. Also, dill extract and essential oils have been used to maintain chemical quality, improve quality sensory properties, and extend the shelf life of seafood. Kannaiyan et al. (2015) showed that due to the antioxidant and antibacterial properties of dill extract boiled in a microwave oven, the shelf life of mackerel fillets stored in the refrigerator was extended by 3 days. Tümerkan et al. (2022) revealed that the use of dill leaves extended the shelf life of tuna paste in terms of sensory quality. Even though several researchers have studied dill extracts and essential oils as a natural and edible coating material for seafood products to enhance quality and extend shelf life, to the best of our knowledge, there are no available reports of the effect of dill extract on the shelf life and quality criteria of fish croquettes.

Based on the above, the aim of this study is (1) to make croquettes from rainbow trout as a ready‐made food product in order to increase the consumption of seafood and (2) to determine the effect of dill extracts obtained by different methods on the quality changes and shelf life of these croquettes during refrigerated storage.

## 2 MATERIALS AND METHODS

A total of 60 rainbow trout (*Oncorhynchus mykiss*) with an average length of 27.13 ± 1.13 cm and a weight of 272 ± 13.1 g were used for making croquettes. Fresh rainbow trout were purchased from a local fish farmer and they were transferred in insulated box containing ice within 1 h to the Fish Processing Technology laboratory. Dill (*Anethum graveolens* L.) was purchased from a local market in Rize, Turkey.

### 2.1 Preparation of extracts

Fresh dill was used in the preparation of the extracts. Two different extraction methods including brewing and distillation were used. Distillation–extraction was carried out as reported by Karsli, Caglak, and Kilic (2021) with some modifications. The distillation–extraction of fresh drill samples was carried out in distilled water in a ratio of 1:5 (w/v) for 30 min by using distillation device (Gerhardt KI 12/24). For the brewing extraction, dill samples were added in boiled distilled water with a ratio of 1:5 (w/v) for 30 min. Then, all extracts were filtered through Whatman No. 1 filter paper for both extraction methods and stored in amber glass bottles in the dark at 4°C until utilization.

### 2.2 Preparation of fish croquettes

**FIGURE 1:**
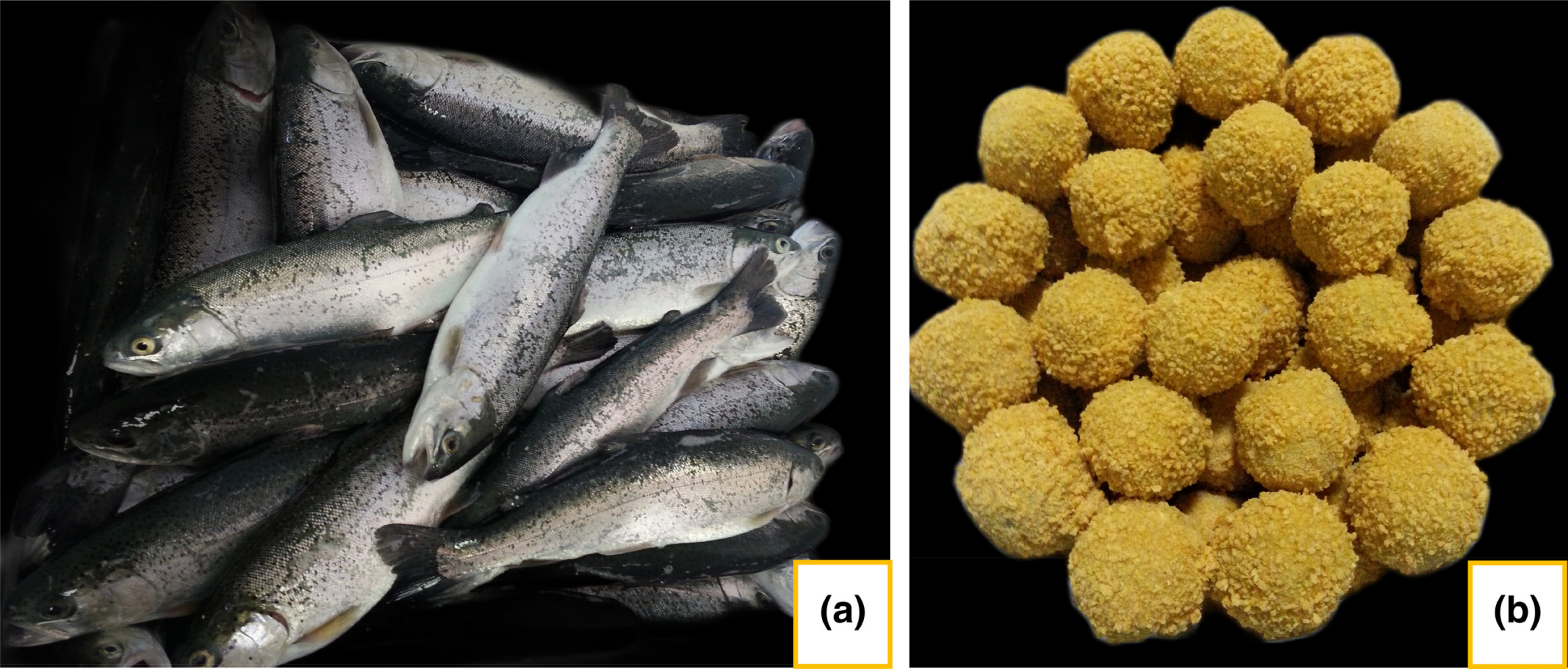
Rainbow trout (a) and croquettes (b) used in this study (original).

The fish, which were cleaned by removing their internal organs, were placed in an oven bag and subjected to a precooking/boiling process for 20 min in a hot water bath heated at 80°C. The skin and bones of the cooked fish were removed, and the net meat amount of the boiled products was determined as 5500 g. After this stage, the croquette preparation process was carried out. For the croquette dough, 96.12% trout meat, 0.85% bread crumbs, 0.43% wheat flour, 0.05% corn starch, 0.32% curry, 0.38% coriander, 0.16% garlic salt, and 0.24% black pepper were added and the ingredients were mixed and homogenized. The formed croquette dough was divided into three groups: (1) control: 1.45% added distilled water; (2) BDA: 1.45% added brewed dill extract; (3) DDA: 1.45% distilled dill extracts. Thus, the ratio of croquette dough was adjusted to 100%. Then, the croquette dough was given a round shape and coated (Figure 1). In the coating process, eight eggs, 75 g yeast, 7.5 g wheat flour, and 30 g commercial granule coating material were used. The prepared croquettes were cooked in the oven at 190°C for 3 min and cooled at room temperature for 5 min. Then, the products were placed on styrofoam plates, wrapped with stretch film, and stored at 2 ± 1°C. Three plates from each croquette group were taken randomly and analyzed every 4 days for 61 days. All analyses were performed in triplicate.

### 2.3 Proximate composition

The moisture content of the croquette was determined by drying it in an oven (ON‐21E, JEIO Tech Co.) at 105°C until a constant weight (AOAC, 1995, Method 985.14). Crude fat content was determined using an automatic Soxhlet analyzer (Velp SER 148/6), and petroleum ether was used as solvent. Crude protein analysis was carried out using Kjeldahl method with an automatic distillation unit S5 (Behr Labor Technik) (AOAC, 1980, Method 2.507). Crude ash content was detected by burning method in a muffle furnace (Şimşek Laborteknik KF‐908) at 550°C (AOAC, 1980, Method 7.009). The proximate composition of the samples was determined in fresh, precooked, and spiced fish, as well as in all study groups on the first day of storage.

### 2.4 Sensory analyses

The sensory assessment of croquette samples was carried out according to the methods reported from Çağlak et al. (2017) with some modifications. The sensory scores including odor, flavor, appearance, texture, and overall acceptance of samples were evaluated using a 9‐points hedonic scale (9–7: very good; 6.9–5.1: good; 5–4: consumable; 3.9–1: unacceptable) by six experienced panelists (40% females and 60% males; aged between 32 and 48 years old) in the sensory evaluation of seafood.

### 2.5 Physicochemical analyses

Determination of total volatile basic nitrogen (TVB‐N) was performed according to the Lücke and Geide method (Varlık et al., 1993). First, 10 g of croquette samples were mixed with 100 mL distilled water and homogenized using a WiseTis HG‐15D homogenizer (Daihan Scientific Co.) for 2 min. The homogenized samples were put in the Kjeldahl tube, then 2 g of magnesium oxide (MgO) and one drop of silicone oil to prevent foaming were added into the Kjeldahl tubes. On the other hand, 100 mL of distilled water, 10 mL of 3% boric acid, and 8 drops of tashiro indicator were added to the 500 mL Erlenmeyer flask where the distillate would be collected. Steam distillation was carried out for 15–20 min using a Kjeldahl distillation unit (Gerhardt KI 12/24). The distillate was titrated with 0.1 M HCl until neutralized and TVB‐N value expressed according to the formula:

$$\mathrm{TVB}-N\;\left(\mathrm{mg}/100\;g\;\text{sample}\right)=V\times 1.4\times 100/W$$

where *V* is the titration volume (mL) of 0.1 mol equi/L HCI used and *W* is the weight of trout croquette (g).

The thiobarbituric acid (TBA) analysis was carried out to determine the degree of lipid oxidation of samples according to the method described by Tarladgis et al. (1960). For TBA analysis, 10 g of croquette samples were mixed with 50 mL distilled water and homogenized using a WiseTis HG‐15D homogenizer (Daihan Scientific Co.) for 2 min. The homogenized samples, 47.5 mL distilled water, and 2.5 mL of 4 M HCI were added in Kjeldahl tubes. The distillation was continued until 200 mL of distillate was collected and 5 mL of distillate and 5 mL of TBA reagent were placed in a capped glass tube and boiled in a water bath for 35 min. The absorbance of the solutions in the tubes cooled at room temperature was measured with a spectrophotometer (Shimadzu UV–vis 1800) at a wavelength of 532 nm. The TBA value was calculated by multiplying the absorbance value by a factor of 7.8 and results were expressed as mg malondialdehyde/kg of samples.

The pH of the croquette was measured using a digital pH meter (Hanna, HI 3220) by making homogeneous mixtures of fish and distilled water (1:10, w:v) (Karsli, Caglak, & Prinyawiwatkul, 2021b). The water activity (*a*_w_) was determined using dew point water activity meter Aqualab Series 4TE (Decagon) at 23–24°C. The color analysis of the homogenized samples was determined using CR‐14 Color Reader (Konica Minolta). The lightness (L* = 0 black to L* = 100 white), redness (+a*)/greens (−a*), and yellowness (+b*)/blueness (−b*) values were determined according to the CIE Lab coordinates.

### 2.6 Microbiological analysis

The total aerobic mesophilic (TAMB) and psychrophilic (TAPB) bacteria counts and total coliforms (TC) test was carried out according to the method recommended by Halkman (2005). Twenty‐five grams of croquette were mixed with 225 mL of physiological saline solution (0.85% NaCl) and the mixture was homogenized using a Bagmixer 400 Stomacher (Interscience) for 2 min. Then, the decimal dilutions were prepared and 0.1 mL of each dilution was inoculated onto Petri dishes containing plate count agar (PCA) for total viable bacteria count (TVC) and violet red bile (VRB) agar for TC. The Petri films of TAMB, TAPB, and TC were incubated at 37°C for 48 h, 7°C for 10 days, and 35°C for 24 days, respectively. Microbial counts were expressed as log CFU/g.

### 2.7 Statistical analyses

All analyses were performed in triplicate and the results were expressed as mean values with standard deviations. A one‐way ANOVA (analysis of variance) method followed by the smallest significant difference (LSD) test was used to determine the differences among the treatments at *p* < .05 using JMP software (SAS Institute, Inc.; Sokal & Rohlf, 1987).

## 3 RESULTS AND DISCUSSION

### 3.1 Proximate composition

The crude protein, crude fat, moisture, and crude ash values of the C, BDA, and DDA groups during refrigerated storage are given in Table 1. The protein content, which was determined as 18.48% in fresh trout, was found to be 20.78% and 22.05%, respectively, after precooking (PT) and spice additive (ST) processes. The protein values of trout croquettes were 20.13% in the C group, 22.05% in the BDA group, and 19.61% in the DDA group on first day of storage. Changes in protein content were found to be statistically insignificant among groups (*p* > .05). Crude fat values were found to be 5.51% in the fresh trout, and this value increased after precooking (6.25%) and spice additive (6.96%) processes (*p* < .05). Crude fat values of C, BDA, and DDA groups were 11.37%, 11.53%, and 10.19%, respectively. No statistically significant difference was found among the fat contents of the croquette groups (*p* < .05). Moisture content was found as 76.70% in the fresh. Depending on the precooking process, the trout lost water and the moisture content decreased to 72.47% (*p* > .05). Moisture contents of ST, C, BDA, and DDA were found to be significantly lower compared to fresh (*p* < .05). However, no significant differences were detected among the croquette groups (*p* > .05). The ash content found as 1.40% in the fresh sample did not show any significant (*p* > .05) changes despite the applied treatments and this value was determined in the range of 1.44%–1.71%. Similarly, Karsli, Caglak, and Kilic (2021) reported the protein, fat, moisture, and ash contents of raw rainbow trout were 18.63%, 5.48%, 75.32%, and 1.18%, respectively. Çankırılıgil and Berik (2017) determined the protein, fat, moisture, and ash amounts in fresh rainbow trout to be 15.47%, 4.11%, 77.78%, and 1.94%, respectively. In addition, these researchers found these values to be 13.77%, 6.48%, 58.13% and 3.01% in croquette trout, respectively. The overall biochemical composition of fish may vary depending on the species, age, height, sex, diet, environmental conditions, and processing methods (Cağlak & Karsli, 2013). In this study, the amount of moisture in the raw rainbow trout decreased in all croquette groups. This situation is caused by the removal of water by the additives (salt and spices) used together with the heat treatment applied while processing fish meat. Similar situations were observed in studies carried out on croquettes from rainbow trout (Berik et al., 2011), fish ball (Özpolat & Çoban, 2012), and fish burgers (Taşkaya et al., 2003) obtained from different fish species.

**TABLE 1:** Proximate composition of rainbow trout croquettes (%).

	Groups	Crude protein	Crude fat	Moisture	Crude ash
	F	18.48 ± 1.40^a^	5.51 ± 0.01^c^	76.70 ± 0.60^d^	1.40 ± 0.14^a^
	PT	20.78 ± 2.06^a^	6.25 ± 0.01^b^	72.47 ± 1.07^c,d^	1.44 ± 0.01^a^
	ST	21.05 ± 2.25^a^	6.96 ± 0.04^b^	69.03 ± 0.11^c^	1.57 ± 0.03^a^
Day 1	C	20.13 ± 1.08^a^	11.37 ± 0.01^a^	53.08 ± 1.74^b^	1.54 ± 0.03^a^
	BDA	22.05 ± 1.12^a^	11.53 ± 0.16^a^	41.80 ± 0.27^a^	1.71 ± 0.01^a^
	DDA	19.61 ± 0.24^a^	10.19 ± 0.20^a^	51.64 ± 3.77^b^	1.66 ± 0.04^a^

*Note*: Different superscripts (^a–d^) in the same column indicate significant differences (*p* < .05) among groups.

Abbreviations: BDA, brewed dill extract added group; C, control; DDA, distilled dill extract added group; F, fresh; PT, precooked trout; ST, spiced trout.

### 3.2 Sensory parameters

**FIGURE 2:**
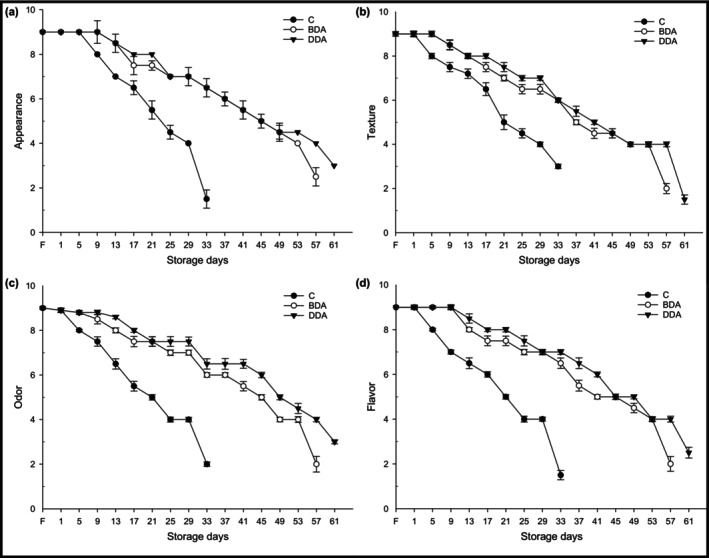
Sensory scores (appearance, texture, odor, and flavor) of trout croquettes during refrigerated storage. BDA, brewed dill extract added group; C, control; DDA, distilled dill extract added group; F, fresh.

**FIGURE 3:**
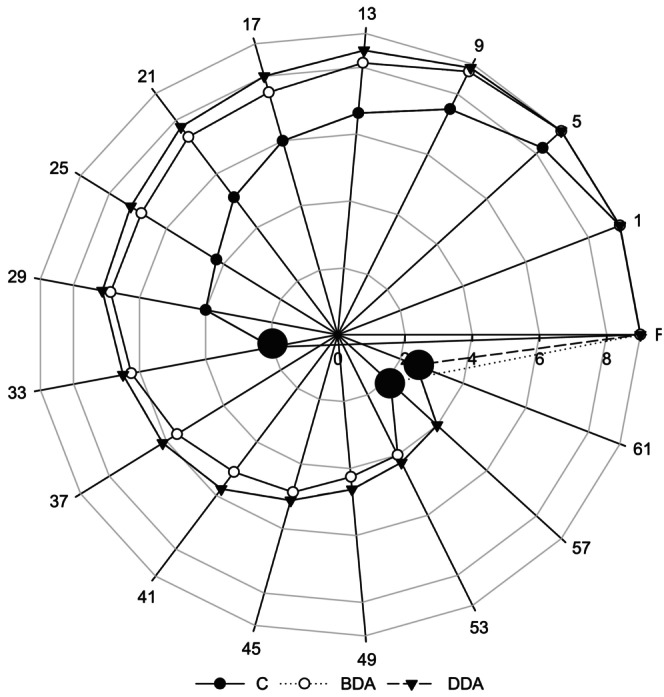
Overall sensory scores of trout croquettes during refrigerated storage. BDA, brewed dill extract added group; C, control; DDA, distilled dill extract added group; F, fresh.

Sensory evaluation results (appearance, odor, flavor, and texture) of trout croquette samples are given in Figure 2. Sensory scores evaluated according to the 9‐point hedonic scale declined consistently in all groups depending on the storage period (*p* < .05). Intragroup change in the decreasing scores of all sensory criteria during refrigerated storage was significant in all three croquette groups (*p* < .05). According to sensory evaluation results, all trout croquette groups were generally liked by consumer groups at the beginning of the refrigerated storage. However, compared to the dill extract‐treated groups, the scores of the control group were statistically lower from day 13 for appearance and from day 9 for odor, taste, texture, and general sensory scores (*p* < .05). Considering the acceptability limit of 3.9 points, the overall sensory scores of the croquette groups were below the consumable limit value on the 33rd day in the control group, on the 57th day in the BDA, and on the 61st day in the DDA (Figure 3). The use of dill extract extended the shelf life of trout croquettes by 24 days for BDA and 28 days for DDA compared to the control group.

In this study, the results showed that dill extract additive increased the sensory properties and quality of croquette products and could be used as a natural antioxidant and antimicrobial additive in seafood products. Therefore, treatment with dill extracts has a potential to improve the storage quality of foods such as aquaculture. Similarly, Tümerkan et al. (2022) reported that the use of dill leaves in tuna paste preserves the sensory quality of the tuna paste and prolongs its shelf life. In another study, it was reported that fillets treated with dill extracts boiled in a microwave oven had higher sensory scores compared to the control group (Kannaiyan et al., 2015). Keser and İzci (2020) reported that the addition of rosemary and laurel essential oils to fish balls from rainbow trout had a slightly positive effect on overall acceptability terms of sensory evaluation and that all groups remained within the consumable limit values during the 14‐day cold storage period. It has also been reported that many different plant extracts protect the sensory quality of fish and its products (Balıkçı & Yavuzer, 2018; Bensid et al., 2014; Özyurt et al., 2012; Ucak & Fadıloğlu, 2020). Çankırılıgil and Berik (2017) reported that the croquettes obtained from rainbow trout were appreciated by the panelists in their very good class and remained within the sensory consumable limit values during the 32‐day cold storage period. Berik et al. (2011) reported the sensory analysis results of the croquettes obtained from rainbow trout fillets as very good according to the sensory scale used. Çankırılıgil and Berik (2018) stated that croquettes from different seafood products (rainbow trout, sardine, and shrimp) were appreciated by both 100 consumers and 12 expert panelists, according to sensory taste tests.

### 3.3 Chemical and physical quality parameters

**FIGURE 4:**
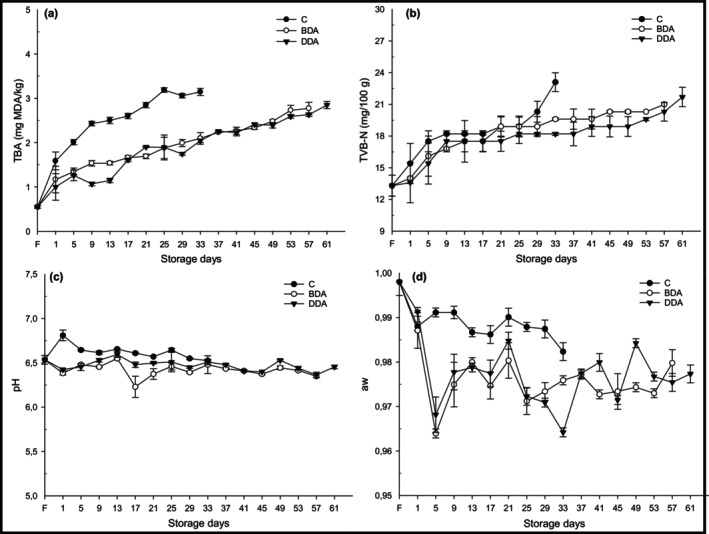
Changes in TBA (a; mg MDA/kg), TVB‐N (b; mg/100 g), pH (c), and *a*_w_ (d) of trout croquettes during refrigerated storage. BDA, brewed dill extract added group; C, control; DDA, distilled dill extract added group; F, fresh.

TBA, TVB‐N, pH, and water activity results of refrigerated fish croquette are given in Figure 4. Fish are highly exposed to oxidation due to their high level of unsaturated fatty acids compared to other animal fats (Al‐Saghir et al., 2004).

Lipid oxidation causes off‐flavor, rancidity, and discoloration. In this respect, determination of lipid oxidation is an important step in determining the freshness and quality criteria of seafood products. TBA analysis is widely used as an important indicator in the determination of lipid oxidation in seafood (Karsli, Caglak, & Prinyawiwatkul, 2021a). In this study, the initial TBA value of fresh rainbow trout fillet was 0.54 mg MDA/kg and this value increased significantly (*p* < .05) to 3.13, 2.77, and 2.85 mg MDA/kg for C (33th day), BDA (57th day), and DDA (61st day) groups, respectively, at the end of refrigerated storage (Figure 4a). TBA values of all croquette groups did not exceed the consumable limit value of 5 mg MDA/kg recommended by Sallam (2007). However, dill extract showed an antioxidant effect on fish croquettes, and the TBA value of C was found to be higher compared to the dill extract added samples (*p* < .05) during storage. There was no significant difference in TBA values between the BDA and DDA groups (*p* > .05). The increase in TBA value on the first day of cold storage has thought to be due to the temperature and additives applied in the croquette preparation steps. The lower TBA values of croquette treated with dill extracts may be attributed to the antioxidant components such as phenols, flavonoids, and proanthocyanidins (Shyu et al., 2009). Singh et al. (2005) reported that dill extract could be an alternative source of natural antioxidants. Similar findings were reported that dill extracts were applied to limit lipid oxidation in seafood. Kannaiyan et al. (2015) reported that dill extract obtained by boiling in a microwave oven extended the shelf life of mackerel fillets by 3 days and that these natural antioxidant‐containing extracts could be an effective alternative to synthetic antioxidant substances. Çankırılıgil and Berik (2017) found that TBA values of trout croquette during 32 days of cold storage were between 0.89 and 1.63 mg MDA/kg. In another study, it was reported that the TBA values increased during the processing stages of the fish croquettes obtained from *Barbus esocinus* and varied between 0.70 and 3.73 mg MDA/kg during the 21‐day storage period (İnanlı et al., 2006). These lower TBA values were in agreement with our study results.

The initial TVB‐N value of rainbow trout was 13.30 mg/100 g, and TVB‐N value of all croquette groups increased significantly (*p* < .05) according to storage time (Figure 4b). TVB‐N value of fresh rainbow trout is consistent with the values (14.08 mg/100 g) reported by Çağlak and Karslı (2015). In this study, dill extracts showed a delaying effect on the increase of TVB‐N values, and TVB‐N values of croquette groups (BDA and DDA) were found significantly lower over storage time (*p* < .05). Especially, croquette group (DDA) treated with dill extract obtained by distillation method was found lower compared to other groups (*p* < .05). Even during 61 days storage period, the TVB‐N value of DDA did not exceed the acceptability limit of 30 mg/100 g recommended by Connell (1975). The lower TVB‐N values of croquette treated with dill extracts may be attributed to the antioxidant components such as phenols, flavonoids, and proanthocyanidins (Shyu et al., 2009). Microorganisms play an active role in the increase of TVB‐N values (Fan et al., 2009). In this study, lower bacterial growth in croquette treated with dill extracts was in agreement with the TVB‐N results. Similarly, Çankırılıgil and Berik (2017) reported that the TVB‐N value of trout croquettes during 32 days of cold storage did not exceed the limit value and was 15.54 mg/100 g at the end of storage. Also, Mol et al. (2002) reported that the TVB‐N (19.66 mg/100 g) and pH (5.6) values of fish burgers obtained from rainbow trout stored at +4°C for 28 days remained very low even at the end of storage, and these criteria were not suitable for determining the quality of trout burgers.

At the beginning of the storage, pH value of fresh trout was 6.53 (Figure 4c). The pH value after precooking and spice additive processes was found as 6.43 and 6.79, respectively, and these changes were found as statistically significant (*p* < .05). During the refrigerated storage periods, fluctuations were observed in the pH values of the croquette products. Dill extract did not show much effect on the pH of the croquettes, however; pH of the dill extract added samples was consistently lower than that of control samples (*p* < .05). The lower pH values recorded for dill extract‐treated croquettes are confirmed by the antimicrobial effect of dill extracts, inhibiting bacteria that cause protein breakdown and the production of key compounds responsible for the pH increase. The low pH value in croquettes treated with dill extract is probably due to the pH stabilizing property of dill extract (Tizghadam et al., 2021). Similarly, it has been reported by some researchers that various herbal extracts do not have much effect on the pH of fish products during storage (Çağlak & Karslı, 2016; Duman et al., 2012; Karsli, Caglak, & Kilic, 2021; Selmi & Sadok, 2008).

Water activity (*a*_w_) is an important factor in controlling microbial activity and in terms of organoleptic quality parameters (Karsli, Caglak, & Kilic, 2021). In this study, the *a*_w_ value, which was 0.9980 in fresh rainbow trout, decreased to 0.9931 with the precooking process, and then to 0.9883 with the spice addition process (Figure 4d). During the storage, fluctuations in *a*_w_ values of all croquette groups were observed; however, the *a*_w_ value of control was found statistically higher than those treated with dill extracts (*p* < .05). During the study, the *a*_w_ ranged between 0.98 and 0.99 in the control (C) group and 0.96 and 0.97 in the BDA and DDA groups. Similarly, it was stated that the *a*_w_ values of the fish croquettes made from *Barbus esocinus* were between 0.95 and 0.98 during the 21‐day cold storage period (İnanlı et al., 2006). Karsli, Caglak, and Kilic (2021) stated that the *a*_w_ values of rainbow trout treated with green tea and black cumin ranged from 0.9877 to 0.9948 during the refrigerated storage period. It is thought that the slight decreases observed in water activity values compared to fresh products during storage are the result of the applied precooking and spice addition processes, as well as the deterioration of the connective tissue of the fish meat.

**FIGURE 5:**
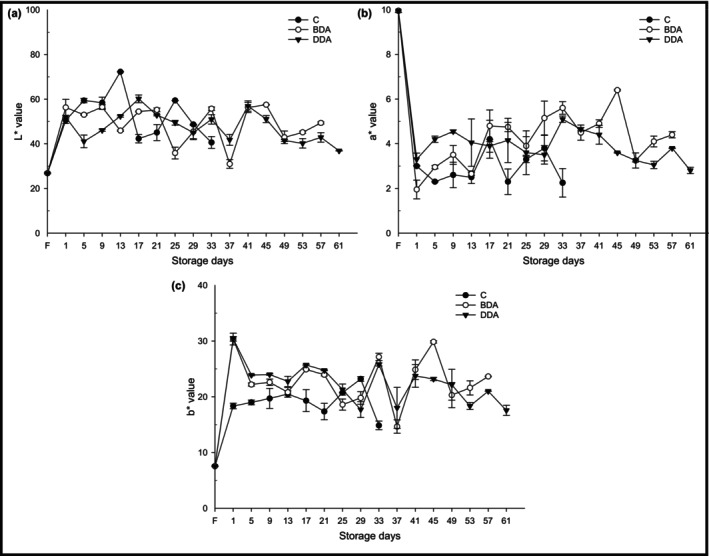
Changes in the color (L*, a*, and b*) of trout croquettes during refrigerated storage. BDA, brewed dill extract added group; C, control; DDA, distilled dill extract added group; F, fresh.

Color, which is one of the sensory evaluation criteria and an important parameter in terms of acceptability of consumers, can directly affect the purchasing decision of consumers. According to the results of the color analysis, the L* (26.80), a* (9.95), and b* (7.55) values were found to be 26.80, 9.95, and 7.55, respectively (Figure 5). In all croquette groups, on the first day of storage after the precooking and the spice addition processes, L* and b* value increased significantly (*p* < .05), while a* value was decreased significantly (*p* < .05). A fluctuating change in L*, a*, and b* values of the croquette groups was observed throughout the refrigerated storage. Also, the dill extract treatments did not have a significant effect on the L*, a*, and b* values of the croquettes compared to the control group. However, it was thought that the significant changes observed on the first day of storage were caused by both precooking and adding spice processes. Karsli, Caglak, and Kilic (2021) reported an increase in L* and a* values and a decrease in b* value of rainbow trout fillets treated with black cumin and green tea extracts on the first day of refrigerated storage. In another study, it was reported that L* and a* values decreased and b* values increased during the 7‐day cold storage period of sardine patties (Kilinc et al., 2008). These changes observed in the color values of this study may have resulted from different plant extracts, methods, and fish species.

### 3.4 Microbiological parameters

**FIGURE 6:**
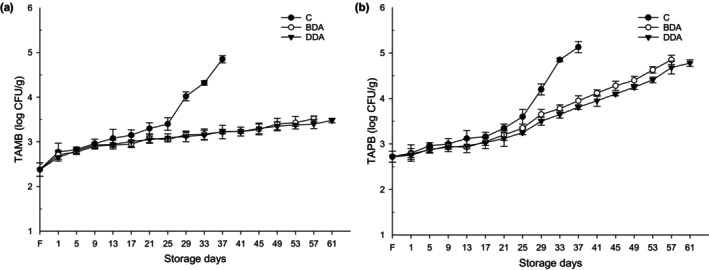
Changes in TAMB (a) and TAPB (b) of trout croquettes during refrigerated storage. BDA, brewed dill extract added group; C, control; DDA, distilled dill extract added group; F, fresh; TAMB, total aerobic mesophilic bacteria; TAPB, total aerobic psychotropic bacteria.

The changes in TC and TVC including TAMB and TAPB in trout croquettes were analyzed during the refrigerated storage, and TVC results were presented in Figure 6. In fresh samples, the TAMB, TAPB, and TC were determined as 2.38 log CFU/g, 2.72 log CFU/g, and 2.04 log CFU/g, respectively. These all counts decreased after the precooking process of the products and were found to be below the detectable limit (1.47 log CFU/g). TAMB of croquette groups increased significantly (*p* < .05) during the refrigerated storage and reached 4.85, 3.52, and 3.48 log CFU/g in the C, BDA, and DDA, respectively, at the end of the storage period. The count of TAPB increased more than the count of TAMB and it was determined as 5.13 log CFU/g in C, 4.85 log CFU/g in BDA, and 4.78 log CFU/g in DDA at the end of each group's storage. During entire storage period, the TC values of croquette groups were found below the detectable limit values, and therefore all data were not shown due to low values of TC. The croquettes treated with dill extract had lower TVC compared to the control samples during the entire storage, indicating that dill extracts, especially DDA, showed an inhibitory effect against total viable bacteria growth. In addition, the TVC values of the BDA and DDA groups were found to be statistically lower than the value of the control group on the 57th and 61th days, respectively (*p* < .05). According to these results, all groups without exception did not exceed the acceptability upper limit value of 7 log CFU/g recommended by ICMSF (1986). Similarly, Çankırılıgil and Berik (2017) reported that rainbow trout croquettes did not exceed these limits during 32 days of refrigerated storage (+4°C), and TAMB (3.3 log CFU/g in fresh) and TAPB (1.5 log CFU/g in fresh) counts reached 6.6 log CFU/g and 3.4 log CFU/g, respectively, at the end of storage. However, compared to our study data, the higher TAMB and lower TAPB counts in the results of Çankırılıgil and Berik (2017) are thought to be due to the initial bacteria loads of the fish and the applied treatments. In this study, the low values in croquettes treated with dill extract can be attributed to the antimicrobial activity of the dill extract (Oshaghi et al., 2015). The high carvone and limonene content may have played an important role in the antimicrobial mode of action of dill (Mutlu‐Ingok & Karbancioglu‐Guler, 2017). The reason for the restriction in TVC can also be attributed to the use of crude extracts containing flavonoids in the glycosidic form, where the sugar in the extracts reduces activity against some bacteria (Bensid et al., 2014). In addition, some studies have reported antimicrobial effects of dill extract and essential oils against various microorganisms (Jianu et al., 2012; Mutlu‐Ingok & Karbancioglu‐Guler, 2017; Singh et al., 2005).

## 4 CONCLUSION

In conclusion, the dill extracts obtained by both brewing and distillation methods showed antioxidant and antimicrobial effects on trout croquettes, which can be consumed as healthy fast food. None of the croquette groups exceeded the limit values both chemically and microbiologically at the end of the refrigerated storage, however; the extract groups showed lower chemical and microbiological values compared to the control group. The sensory evaluation indicated that the shelf life of trout croquettes was 33 days for C group, 57 days for BDA group, and 61 days for DDA group. The results showed that the addition of dill extract improved the sensory and microbial quality of fish and extended the shelf life by 24–28 days compared with the control samples. Therefore, it can be suggested that the distillation method would be ideal for the extraction of dill. It has been concluded that dill extract additives can be used as a natural preservative with antioxidant and antimicrobial properties on products such as croquettes, hamburgers, meatballs, and sausages that can be produced from fish. Thus, the extract treatment could have a wide application prospect in the industrial processing and storage of seafood. In addition, it was foreseen that increasing the shelf life of such products would have important effects in terms of healthy nutrition, practical consumption habits, product variety, and sales and marketing network.

## AUTHOR CONTRIBUTIONS

**Emre Caglak:** Conceptualization (equal); investigation (equal); supervision (lead); writing – review and editing (equal). **Barış Karsli:** Conceptualization (equal); data curation (lead); formal analysis (lead); investigation (equal); visualization (lead); writing – original draft (lead).

## CONFLICT OF INTEREST STATEMENT

The authors declare that they have no conflict of interest.

## Data Availability

Data will be made available on request.
